# Invertible Privacy-Preserving Adversarial Reconstruction for Image Compressed Sensing

**DOI:** 10.3390/s23073575

**Published:** 2023-03-29

**Authors:** Di Xiao, Yue Li, Min Li

**Affiliations:** College of Computer Science, Chongqing University, Chongqing 400044, China

**Keywords:** compressed sensing, image reconstruction, privacy preserving, adversarial examples, invertible

## Abstract

Since the advent of compressed sensing (CS), many reconstruction algorithms have been proposed, most of which are devoted to reconstructing images with better visual quality. However, higher-quality images tend to reveal more sensitive information in machine recognition tasks. In this paper, we propose a novel invertible privacy-preserving adversarial reconstruction method for image CS. While optimizing the quality, the reconstructed images are made to be adversarial samples at the moment of generation. For semi-authorized users, they can only obtain the adversarial reconstructed images, which provide little information for machine recognition or training deep models. For authorized users, they can reverse adversarial reconstructed images to clean samples with an additional restoration network. Experimental results show that while keeping good visual quality for both types of reconstructed images, the proposed scheme can provide semi-authorized users with adversarial reconstructed images with a very low recognizable rate, and allow authorized users to further recover sanitized reconstructed images with recognition performance approximating that of the traditional CS.

## 1. Introduction

With the rapid development of the Internet of Things, the scale of the network becomes larger and larger, and the network environment becomes more and more complex. In the Internet of Things, the number of smart wireless sensors has increased significantly, which has brought great challenges to network communication. Problems such as energy saving, transmission efficiency, and security have gradually attracted attention. Compressed sensing (CS) [1] can help to solve the three problems of intelligent network communication simultaneously. CS is an advanced signal sampling and reconstruction method, which breaks the Nyquist sampling theorem. It can approximately restore the original signal through a few measurements, and its speed of sampling is much faster than similar traditional frameworks. It is usually used as a data encryption and compression scheme for energy-constrained wireless sensor networks.

Over the past decades, many classic traditional CS reconstruction algorithms have been proposed [2,3,4], such as the orthogonal matching pursuit algorithm (OMP) [2] and the gradient projection for sparse reconstruction algorithm (GPSR) [3]. However, such traditional reconstruction algorithms often have high computational costs, and when the sampling rate is low, it is usually difficult to obtain a reconstructed image with good quality.

Fortunately, the emergence of deep learning provides new possibilities for the reconstruction of CS. In recent years, deep learning, as a hot technology in the era of big data, has been successfully applied to the field of computer vision and has made a series of breakthroughs in tasks such as image recognition, super-resolution, and image restoration. The problem that the sparse hypothesis model in traditional CS cannot fully meet the application requirements can be solved by introducing deep learning. The CS methods based on deep learning [5,6,7] not only improve the reconstruction performance but also complete image reconstruction in a very short time, meeting the real-time requirements in real-world applications.

Most of the existing CS-related works based on deep learning are dedicated to improving the visual quality of reconstructed images, and the security they are concerned about is usually reflected in whether illegal users can restore the image content with the measurements. In fact, after the receiver has recovered high-quality reconstructed images, the privacy threat still is living. In the past, if the image content could not be recognized by human eyes, we said the image’s privacy was protected. However, in the era of big data, reconstructed images may be collected by unauthorized third parties, and then intelligent algorithms may be maliciously used for data analysis or model training, which poses a great threat to the privacy of image owners. Therefore, we propose a privacy-preserving requirement for practical applications: we hope that the reconstructed image can only meet the observation needs of the receiver’s eyes, but it cannot be used for machine tasks successfully.

From the perspective of an individual, Kashmir from the New York Times reported on Clearview.AI, a company that has collected more than 3 billion online photos and trained a large model to identify millions of citizens. However, the company finished the collection without the knowledge or explicit consent of the photo owners [8], which poses a huge threat to personal privacy. In response to this threat, Ref. [9] suggests that users add an imperceptible “cloak” perturbation before uploading their photos to social networks. As a result, the facial recognition model would make wrong judgments. From the perspective of the companies, they also want to prevent valuable internal data from being stolen by malicious employees for analyzing data or training pirated models because both of these behaviors may cause huge economic or reputational losses to the company.

Taking the above scenarios as examples, therefore, we hope that the defensive version of the reconstructed image can be viewed normally by human users. However, if receivers try to collect a large number of reconstructed images without authorization to train the pirate model, it is impossible for them to obtain a model with good performance. Of course, the receivers cannot obtain accurate and reliable privacy information by analyzing the defensive version of the reconstructed images. That is, although reconstructed images have good visual quality, they cannot be used for effective training or accuracy recognition.

This motivation reminds us of adversarial examples [10]. Nowadays, adversarial examples are a research hotspot in the security field of artificial intelligence, which aim to deceive deep neural network (DNN) models by adding imperceptible perturbations to the original samples. We use this double-edged sword in a defensive way to preserve the privacy of CS reconstructed images.

Destroying the availability of reconstructed images for machine tasks is very costly, as huge amounts of data are essential to building a well-performing deep learning model. However, both collecting data and labeling it are costly tasks, which are time-consuming and labor-consuming. Therefore, datasets are often viewed as digital property, and some companies offer them to users as paid content. If reconstructed images are simply transformed into images that cannot be trained or recognized, authorized users will lose the right to use the data properly, which causes unnecessary waste of resources. Thus, it is imperative for authorized users to restore the privacy-preserving reconstructed images to clean samples that can be used for training and recognition effectively. In other words, the adversarial reconstructed images should be reversible [11]. A reversible privacy-preserving framework for face recognition is also proposed in [12].

Inspired by [13], we pay more attention to the performance of reconstructed images in machine tasks. However, our goal is not to improve the recognition performance of reconstructed images but to achieve privacy protection by reducing their recognition accuracy. Therefore, we propose a novel invertible privacy-preserving adversarial reconstruction method for image CS based on adversarial examples (IPPARNet). We divide the users into two categories: semi-authorized users and authorized users. The adversarial reconstruction network takes the measurements as input and then outputs the reconstructed image, which is an adversarial sample. Such reconstructed images could be obtained by a semi-authorized user. Although they can be recognized normally by human eyes, the DNN models will be misled by them and make incorrect inferences, so as to achieve the purpose of privacy protection. At the same time, we also consider the invertibility of machine task availability. For authorized users, they can employ an additional restoration network to restore the adversarial sample to a sanitized sample which is helpful for machine tasks, avoiding the secondary transmission of available data. The authors of [13] point out that machine users pay more attention to machine metrics such as image recognition accuracy, rather than visual quality. Inspired by this, we also regard recognition accuracy as an extra optimization goal of the authenticated restoration network to improve the recognizability of recovered clean samples.

The main contributions of this paper are summarized below:

- We consider both the visual quality of reconstructed images and their ability to confuse DNN models during the reconstruction process of CS so that the reconstructed images have the ability to fool the DNN models at the time of generation.
- We propose a privacy-preserving reconstruction method for image CS based on adversarial examples for users with two levels. While guaranteeing the visual quality of the reconstructed images, we take the machine recognition metric as the starting point and focus on the privacy needs of different users. We not only follow the original adversarial samples but also consider the invertibility of task availability of reconstructed images. Specifically, semi-authorized users can only obtain adversarial reconstructed images, which protects user privacy by reducing the accuracy rate of the recognition models. In contrast, authorized users can restore sanitized reconstructed images from the adversarial reconstructed images for more efficient model training or more accurate data analysis, enabling invertibility for machine task availability.
- The good performance of the IPPARNet is demonstrated with extensive experiments. Keeping good visual quality, the recognizability of adversarial reconstructed images is low enough to avoid being used illegally by malicious users, while the sanitized reconstructed images can reach an approximate or even slightly higher recognition rate compared with that of the original CS reconstructed images.

The rest of this paper is organized as follows. In Section 2, we review the related work of CS and adversarial examples. In Section 3, we introduce the proposed scheme in detail. Section 4 provides the experimental setting and presents the experimental results. Finally, our work is concluded in Section 5.

## 2. Related Works

### 2.1. Compressed Sensing

#### 2.1.1. Traditional Compressed Sensing

The proposal of CS breaks the Nyquist–Shannon theorem and provides a concise and efficient signal acquisition paradigm. The theory of CS points out that as long as the original signal x$\;\in R^{n}$ is sparse in a certain transform domain, it is possible to project the transformed high-dimensional signal onto a low-dimensional space with a measurement matrix $\Phi \in R^{m\times n}$ that is uncorrelated with the orthogonal transform basis $\Psi \in R^{n\times n}$. Then, by solving an optimization problem, the original signal can be reconstructed with high probability from the measurements $y\in R^{m}$.

The formulation of the CS measurement process can be expressed as:

(1) y=Φx=ΦΨs

where s is the sparse coefficient of the original signal x with respect to the basis Ψ. In addition, m << n and $\frac{m}{n}$ is called the sampling rate. The commonly used measurement matrices include structured random matrix, random Gaussian matrix, random Bernoulli matrix, etc. From Equation (1), it can be seen that the computational complexity of the measurement process of CS is fairly low, which is one of its significant advantages.

The reconstruction of CS can be viewed as the inverse process of measuring, and the original image can be reconstructed by seeking the sparsest solution of Equation (1). Although it is an under-determined problem, it can be converted into a problem of minimizing $L_{0}$ norm due to satisfying the sparsity of the signal, i.e., solving

(2) $$\mathrm{argmin}∥s{∥}_{0}\;\mathrm{subject}\;\mathrm{to}\;y=\Phi \Psi s.$$

However, solving Equation (2) is an NP-Hard problem, so it is usually solved iteratively using an approximation algorithm. The commonly used traditional reconstruction algorithms can be divided into two main categories, one is based on convex optimization class algorithms, such as basis pursuit algorithm (BP), GPSR [3], and ISTA-Net [7]. The second is based on greedy algorithms, such as the matching pursuit (MP) algorithm and OMP [2].

Although traditional CS greatly reduces the computational complexity in the measurement process, its reconstruction cost is very expensive. In practical applications, the computationally complex reconstruction work is usually outsourced to cloud servers with abundant computing resources. However, the heavy computation of traditional reconstruction algorithms has not been improved from the root cause.

#### 2.1.2. Compressed Sensing Based on Deep Learning

With the rapid development of deep learning, DNNs are applied to implement CS, which not only further improves the reconstruction quality, but also significantly increases the reconstruction speed. In 2015, Mousavi et al. [14] introduced deep learning into CS with fully connected networks for the first time and proposed a stacked denoising autoencoder (SDA) to capture the statistical correlation between different elements of the signal, thereby improving the quality of the reconstructed signal. Compared with the fully connected network, the convolutional neural network (CNN) reduces the number of parameters and enhances the model expression ability with mechanisms such as parameter sharing and local connectivity. In [15], Kuldeep Kulkarni et al. combined CS with CNN for the first time, and proposed a non-iterative block CS (BCS) [16] reconstruction network named ReconNet. In [17], after the reconstructed linear map from the measurement, a residual network was introduced to narrow the gap between the initial reconstructed image and the original image, leading to a higher reconstruction quality.

However, the above schemes only consider the reconstruction process and do not involve the measurement process, and they use the same measurement matrices as the traditional CS algorithms. In [18], based on ReconNet, a fully connected layer is used to simulate the measurement process, and an efficient measurement matrix could be adaptively learned. In this way, the measurements retain more image structure information, and complex manual design is avoided at the same time. By jointly training the new network consisting of a fully connected layer and ReconNet, visually better-quality images can be reconstructed. Based on BCS, [19] employs deep CNN to achieve sampling and reconstruction, and also trains the sampling network and the reconstruction network in an end-to-end way to quickly restore the reconstructed image with better quality. The authors of [20] further optimize the learned measurement matrices, and propose a {0, 1}-binary matrix and a {−1, +1}-bipolar matrix, which are more convenient for storage and hardware implementation in practical applications. In addition, residual learning is also introduced for better reconstruction.

As mentioned above, most deep learning-based CS schemes focus on two issues: the first is how to learn an effective measurement matrix; the second is how to reconstruct images with better quality and higher speed. However, Ref. [13] points out that in some scenarios, reconstructed images are not used for human viewing but for tasks conducted by machine users. Therefore, we should pay attention to what metrics the machine users are concerned about, such as recognition accuracy. Different from [13], it takes recognition accuracy as an extra optimization goal for CS reconstruction networks, aiming to further improve the recognition rate while reconstructing. In this paper, although we also focus on recognition accuracy, we hope that the reconstructed image is an adversarial sample as it is generated, which has the innate ability to fool DNN models. Thus, the adversarial reconstructed images can avoid being abused by unauthorized users for data analysis or model training, and play an important role in privacy protection.

### 2.2. Adversarial Examples

In 2013, Szegedy et al. [21] first proposed the concept of adversarial samples. That is, after applying an imperceptible perturbation to the original image, the DNN models will wrongly classify the image with high confidence. Such perturbation is called adversarial perturbation, and the image to which the adversarial perturbation is added is called an adversarial sample.

In terms of the model prediction errors, the adversarial sample attack can be divided into two categories: untargeted adversarial samples and targeted adversarial samples. The former means that the adversarial sample can be misclassified by the model as any class other than its real class, while the latter refers to the adversarial sample that can be misclassified as the wrong class specified by the attacker. In this paper, we focus on untargeted adversarial samples.

In terms of the generation manner, adversarial samples can be divided into the following three categories: gradient-based, optimization-based, and generation-based.

In [22], Goodfellow et al. proposed a fast method for generating adversarial samples based on gradients called the fast gradient sign method (FGSM). The method adds a small perturbation whose elements are equal to the sign of the elements of the gradient of the loss function with respect to the input, and rapidly increases the loss in a single step, so as to deceive the DNN models. Subsequently, the gradient direction-based adversarial sample generation methods have been widely studied, such as the basic iterative method (BIM) [23] and the projected gradient descent (PGD) [24]. The BIM provides more robust adversarial examples by modifying the one-step update of FGSM to a multi-step iteration. While the PGD, using gradient projection, is considered the strongest first-order adversarial attack method available.

In [10], Carlini et al. considered generating adversarial samples as an optimization problem and proposed the Carlini and Wagner attack (C&W) which continuously optimizes the perturbations according to the set optimization decline, thus achieving a more efficient adversarial sample with smaller perturbations.

However, the above algorithms based on iterative optimization generally suffer from high computational cost and slow running speed. In recent years, with the development of generative adversarial networks (GAN) [25], the generation of adversarial samples has become more diverse. Based on GAN, Xiao et al. [26] proposed a fast adversarial perturbation generation method called AdvGAN. The generator G takes the original image x as the input and outputs the adversarial perturbation G(x). Then, the perturbation is superimposed on the original image to obtain the adversarial sample x + G(x). The mutual game between the discriminator and the generator drives the visual similarity of the adversarial samples and the original images. Since this scheme does not require iterative optimization and generates the adversarial sample in a single forward pass at the inference stage, it significantly improves the generation speed of the adversarial sample while guaranteeing the success rate of the attack and the image quality. AdvGAN++ [27], based on AdvGAN, proposes to make full use of the potential features of the original image to generate adversarial samples. In this paper, the adversarial reconstructed image is an adversarial sample generated by the generation-based method.

## 3. Proposed Method

### 3.1. Overview

Suppose $\left(X,Y\right)=\left\{\left(x_{1},y_{1}),(x_{2},y_{2}),\ldots \ldots (x_{N},y_{N}\right)\right\}$ is the original dataset with N images, where $x_{i}$ represents the original image with the serial number of *i*, and $y_{i}$ represents the classification label corresponding to $x_{i}$. The $x_{i}$ is sampled to obtain a measurement vector $m_{i}$, then receivers can reconstruct the approximate image of the original image with $m_{i}$. Similarly, the reconstructed image dataset can be expressed as $\left(X\prime ,Y\prime \right)=\left\{\left(x_{1}^{\prime },y_{1}^{\prime }),(x_{2}^{\prime },y_{2}^{\prime }),\ldots \ldots (x_{N}^{\prime },y_{N}^{\prime }\right)\right\}$. In most cases, we hope the reconstructed image $x_{i}^{\prime }$ to be visually similar to $x_{i}$ as much as possible, pursuing higher visual quality. However, images with high visual quality often bring privacy leakage problems. For example, when $y_{i\;}^{\prime }$=$\;y_{i}$, these reconstructed images can be analyzed by unauthorized models, or a large number of them can be collected by illegal users for model training.

**Figure 1:**
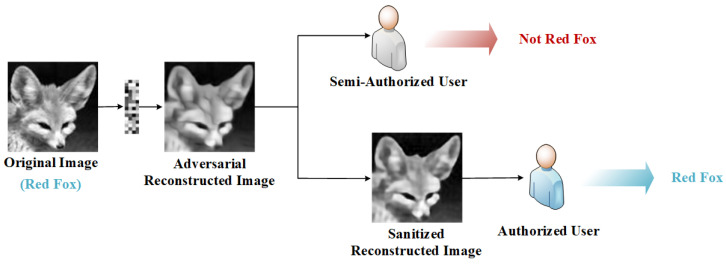
Framework of the proposed method.

Therefore, we propose a novel privacy-preserving adversarial reconstruction framework for CS, as shown in Figure 1. We designed it for semi-authorized users and authorized users. Specifically, the adversarial reconstructed images $x_{i}^{\prime }$, which are reconstructed from the measurement vector $m_{i}$ and could protect the privacy of the original image $x_{i}$, can be obtained by all users.

Our goal is to make $x_{i}^{\prime }$ visually similar to $x_{i}$ as much as possible; however, for the recognition models based on deep neural networks, the corresponding label $y_{i}^{\prime }$ of $x_{i}^{\prime }$ is different from $y_{i}$. That is, the image $x_{i}^{\prime }$ is an adversarial example at the beginning of the reconstruction. In this way, although semi-authorized users obtain visually useful images, they cannot use such images to perform data analysis tasks well, nor can they train effective models. That is to say, while ensuring the practicability of the reconstructed images, we also protect their privacy. When authorized users have task needs, they can restore the adversarial reconstructed image $x_{i}^{\prime }$ to the sanitized image $x{\prime \prime }_{i}$ with the restoration network R. Similarly, for human eyes, the visual difference between $x_{i}^{\prime \prime }$ and the reconstructed image of CSNet should be indistinguishable. However, the classification labels of $x_{i}^{\prime \prime }$ and $x_{i}$ should be the same, that is,$\;y_{i}^{\prime \prime }=y_{i}$. In this way, the sanitized image $x_{i}^{\prime \prime }$ can not only be recognized by human beings, but also be beneficial to machine users for downstream tasks. In this paper, we take the task of recognizing categories as an example. It is worth noting that, inspired by [13], we also pay more attention to the machine users, that is, $x_{i}^{\prime \prime }$ is more helpful to improve recognition performance than the reconstructed image of traditional CS.

### 3.2. Network Architecture

**Figure 2:**
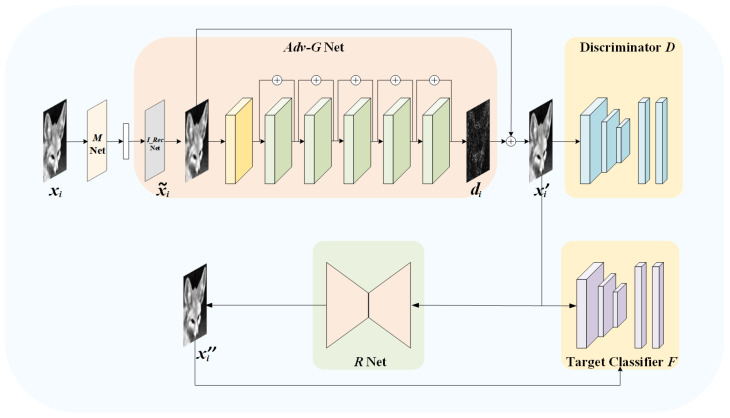
Architecture of the IPPARNet.

Our model framework consists of a measurement network $M_{\Theta_{M}}$, an adversarial reconstruction network Adv-$G_{\Theta_{Adv-G}}$, a discriminator network $D_{\Theta_{D}}$, a restoration network $R_{\Theta_{R}}$, and several pre-trained target classifiers, where the subscripts $\Theta_{M}$, $\Theta_{Adv-G}$, $\Theta_{D}$, and $\Theta_{R}$ are the trained parameters. For simplicity, if there is no ambiguity, we omit the subscripts. Figure 2 shows the overall architecture of our proposed model.

Formally, the measurement process can be expressed as:

(3) $$m_{i}=M\left(x_{i}\right).$$

After the original image $x_{i}$ is measured by the measurement network M, the measurement vector $m_{i}$ is the output.

As for reconstruction, our proposed IPPARNet includes two stages. The first reconstruction stage can be defined as:

(4) $$x_{i}^{\prime }=Adv-G\left(m_{i}\right).$$

With the input $m_{i}$, the Adv−G network outputs the adversarial reconstructed image $x_{i}^{\prime \prime }$. Both networks M and Adv−G are derived from CSNet [20].

We employ several pre-trained classifiers as a joint target network *F*. $x_{i}^{\prime \prime }$ should have the ability to induce the ensemble target network *F* to make wrong inferences while ensuring good image quality. At the same time, we introduce a discriminator *D* to encourage the adversarial reconstructed image $x_{i}^{\prime \prime }$ to achieve a high visual quality.

The second reconstruction stage can be described as:

(5) $$x_{i}^{\prime \prime }=R\left(x_{i}^{\prime }\right).$$

With the additional restoration network R, authorized users can take the adversarial reconstructed image $x_{i}^{\prime \prime }$ as the input, and further restore the sanitized image $x_{i}^{\prime \prime }$. For the target network F, $x_{i}^{\prime \prime }$ can achieve a high recognition accuracy, which is more conducive to subsequent recognition tasks.

#### 3.2.1. Measurement Network: M

Based on BCS, we firstly divide the original image into non-overlapping blocks of size *B* × *B* × *c*, where c represents the number of channels. As shown in Equation (1), the measurement process of traditional CS can be expressed as $m_{i}=\Phi_{B}x_{i}$. Regarding each row of the measurement matrix $\Phi_{B}$ as a filter, the measurement process can be done by a convolutional layer without biases.

For the non-overlapping measurement process, the convolutional layer measures the original image with filters of size *B* × *B* × *c* and a stride of *B*. When the sampling rate is $\frac{M}{N}$, the convolutional layer contains *n* = $\left|\frac{M}{N}cB^{2}\right|$ filters. An image block of size *B* × *B* × *c* is fed into the measurement network to obtain the output measurement vector of size 1 × 1 × *n*. Furthermore, there is no bias in each filter. The measurement network can learn an efficient measurement matrix adaptively, thereby avoiding complicated and possibly inefficient manual design of the measurement matrix.

#### 3.2.2. Adversarial Reconstruction Network: Adv-G

This network can be divided into two components: the initial reconstruction network (referred as I_Rec) and the deep reconstruction network (referred as D_Rec).

In the traditional BCS, the pseudo-inverse matrix is usually used to reconstruct the primary reconstructed image from the measurement value. The implementation of the corresponding network is similar to that of the measurement network. We can also obtain a rough reconstruction of the image with the network I_Rec, which includes a convolutional layer with filters ignoring the bias.

In the I_Rec network, c$B^{2}$ filters of size 1 × 1 × *n* and a stride of 1 can be used to obtain reconstructed image blocks. However, the output of each image block is still a vector at this time, so we need a combination layer to reshape it to a block of size *B* × *B* × *c* and then concatenate these blocks together to obtain the reconstructed image. Since the network I_Rec does not employ any activation layer, the initial reconstruction is a linear operation. The linear mapping produces a relatively good initial reconstructed image ${\tilde{x}}_{i}$ with fast speed and low computational cost, but its visual quality is poor and has obvious block artifacts.

Therefore, we hope to further narrow the gap between ${\tilde{x}}_{i}$ and $x_{i}$ with the network D_Rec. The D_Rec network learns the residual $\;d_{i}=D\_Rec({\tilde{x}}_{i})$, and the final output of the adversarial reconstruction network Adv-G is $x_{i}^{\prime }={\tilde{x}}_{i}+d_{i}$. For the so-called “adversarial reconstruction”, we hope that the learned perturbation $d_{i}$, on the one hand, can further improve the visual quality of the output image of network I_Rec. On the other hand, when it is added to ${\tilde{x}}_{i}$, $x_{i}^{\prime }$ can induce the target network to make wrong predictions, that is to say, $x_{i}^{\prime }$ has the ability to mislead the target model at the moment of generation. For the architecture of D_Rec, we replace residual blocks with those in [28].

#### 3.2.3. Restoration Network: *R*

U-Net [29] is widely used in image processing tasks with DNNs. It uses skip connections to combine the high-level semantic feature maps from the decoder with the corresponding low-level detailed feature maps from the encoder, which is helpful to generate high-quality images. Since the goal of the restoration network R is to obtain the sanitized image $x_{i}^{\prime \prime }$ with the best possible recognition performance while receiving the adversarial reconstructed image $x_{i}^{\prime }$, the network R employs U-net as the backbone. Specifically, its encoder includes four encoding segments, and each segment consists of two convolutions, each of which is followed by a rectified linear unit (ReLU) and a 2 × 2 max pooling operation with stride 2 for down sampling. In addition, each encoding segment doubles the number of feature channels. Correspondingly, the decoder also consists of four decoding segments. Each decoding segment contains a deconvolution layer with stride 2, a connection with the corresponding feature maps from the encoding segment, and 2 convolution layers, each followed by a ReLU. Moreover, each decoding segment reduces the number of channels by half. Finally, the output image of the decoder has the same size as the original image.

#### 3.2.4. Discriminator: D

Our discriminator D is designed as a common 4-layer CNN which outputs a value from 0 to 1 by the sigmoid function. It is used to distinguish between the original image $x_{i}$ and the adversarial reconstructed image $x_{i}^{\prime }$ generated by the network Adv-G. After the continuous game between the network Adv-G and D, $x_{i}^{\prime }$ could have the better visual quality.

#### 3.2.5. Ensemble Target Networks: F

We select three classic classifiers and integrate them as our target network F, which include VGG16 [30], ResNet-50 [28], and DenseNet-121 [31]. Then, we train them on the clean Tiny-ImageNet dataset.

### 3.3. Loss Functions

As mentioned before, we represent the original dataset as $\left(X,\;Y\right)=\left\{\left(x_{1},y_{1}),(x_{2},y_{2}),\ldots \ldots (x_{N},y_{N}\right)\right\}$, where $x_{i}$ represents the original images with the serial number of *i*, and $y_{i}$ represents the classification label corresponding to $x_{i}$.

$L_{G}$**:** The loss function $L_{G}$ of network Adv-G mainly includes four components: the reconstruction loss $L_{G-rec}$, the adversarial loss $L_{G-adv}$, the perception loss $L_{G-per}$, and the classification loss $L_{G-cls}$.

In order to make the output image $x_{i}^{\prime }$ of the network Adv-G similar to the original image $x_{i}$ on the pixel level, following most deep learning-based image restoration methods, we use the $L_{2}$ norm between $x_{i}^{\prime }$ and $x_{i}$ to constrain the difference of them. For the reconstruction loss, we have

(6) $$L_{G-rec}={\left|\left|G\left(M\left(x_{i}\right)\right)-x_{i}\right|\right|}_{2}^{2}.$$

To further narrow the difference between $x_{i}^{\prime }$ and $x_{i}$, we employ the idea of generative adversarial network and introduce the discriminator D. With the help of the discriminator *D*, the network Adv-G is trained in an adversarial way. The adversarial loss can be described as:

(7) $$L_{G-adv}=\log\left(1-D\left(G\left(M\left(x_{i}\right)\right)\right)\right).$$

Furthermore, we introduce the perceptual loss $L_{G-per}$ [32], which is based on feature extractors of VGG16, to optimize the similarity of $x_{i}^{\prime }$ and $x_{i}$ in the feature space. The perception loss can be express as:

(8) $$L_{G-per}=\frac{1}{CHW}{\left|\left|\emptyset_{j}\left(x_{i}^{\prime }\right)-\emptyset_{j}\left(x_{i}\right)\right|\right|}_{2}^{2}.$$

Specifically, we calculate the Euclidean distance between feature maps of $x_{i}^{\prime }$ and $x_{i}$ from the second max-pooling layer. That is, *j* of $\emptyset_{j}$ in Equation (8) is 2.

We take the visual quality and recognition metrics into account at the same time. However, unlike the goal of [13], while they try to improve the recognition performance of the reconstructed images, we attempt to make the adversarial reconstructed images mislead the target network. Therefore, we use the negative cross-entropy to encourage $x_{i}^{\prime }$ to be classified wrongly by the ensemble target networks F. We take the average loss of the three target classifiers as the final classification loss $L_{G-cls}$,

(9) $$L_{G-cls}=-L_{ce}\left(F\left(G\left(M\left(x_{i}\right)\right)\right),y_{i}\right),$$

where $L_{ce}$ is the cross-entropy loss function, $L_{ce}\left(\hat{y},y\right)=-\sum^{\;}y_{i}\log{\hat{y}}_{i}$, which is used to calculate the cross entropy between the predicted label $\hat{y}$ and the ground truth y.

In summary, the total loss of the adversarial reconstruction network Adv-G is defined as follows:

(10) $$L_{G}=L_{G-rec}+g_{1}\cdot L_{G-adv}+g_{2}\cdot L_{G-per}+g_{3}\cdot L_{G-cls},$$

where $g_{1}$, $g_{2}$, and $g_{3}$ are hyper-parameters that play a very significant role in the training process.

$L_{D}$: The same as in [25], the discriminator D is used to distinguish whether the image is the original one or the reconstructed one, and its loss is:

(11) $$L_{D}=-logD\left(x_{i}\right)-\log\left(1-D\left(G\left(M\left(x_{i}\right)\right)\right)\right).$$

$L_{R}$: The goal of the restoration network R is to output the restoration image $x_{i}^{\prime \prime }$ that is not only visually similar to the original image $x_{i}$, but also beneficial for machine recognition tasks. We still use the $L_{2}$ norm to optimize $x_{i}^{\prime \prime }$,

(12) $$L_{R-rec}={\left|\left|R\left(x_{i}^{\prime \prime }\right)-x_{i}\right|\right|}_{2}^{2}.$$

To facilitate the correct recognition of the sanitized image $x_{i}^{\prime \prime }$ by the ensemble target networks, we introduce the positive cross-entropy loss $L_{R-cls}$,

(13) $$L_{R-cls}=L_{ce}\left(F\left(G\left(M\left(x_{i}\right)\right)\right),y_{i}\right).$$

Similarly, we take the average loss of the three target classifiers as the final classification loss. The loss function of the restoration network *R* can be expressed as:

(14) $$L_{R}=L_{R-\mathrm{rec}}+r_{1}\cdot L_{R-\mathrm{cls}},$$

where $r_{1}$ is a hyper-parameter.

In summary, the total loss of the proposed IPPARNet is defined as follows:

(15) $$L=L_{G}+\alpha \cdot L_{R},$$

where α is a hyper-parameter.

### 3.4. Training and Inference

In the training process of the proposed IPPARNet, we alternately train the discriminator D, the adversarial reconstruction network Adv-G, and the restoration network R. Specifically, we optimize the discriminator D by minimizing Equation (11) and then optimize the total loss L. In this way, both the adversarial reconstruction network Adv-G and the restoration network R can generate images with good visual quality, but the former hinders the prediction of the recognizer while the latter can increase the recognition accuracy.

In the inference process, there is no need to use the discriminator D. Feeding the measurement vector $m_{i}$ into the adversarial reconstruction network Adv-G, the adversarial reconstructed image $x_{i}^{\prime }$ is obtained, which can be comprehended by semi-authorized users with human eyes. However, for machine users, $x_{i}^{\prime }$ cannot be recognized accurately, nor can it be used for meaningful training. Therefore, even if malicious users collocate a mass of adversarial reconstructed images, they cannot analyze them effectively or use them to train a model with good performance. However, when the authorized user has task needs, $x_{i}^{\prime }$ can be fed into the recovery network R to output the sanitized image $x_{i}^{\prime \prime }$ that can improve the performance of the machine users in the recognition tasks.

It can be seen that the proposed method mainly introduces a discriminator D and a restoration network R to the original CSNet, achieving our goal by designing loss functions carefully. That is to say, there is no need to change the related hardware for the measuring and reconstructing of the original CS.

## 4. Experiment and Results

### 4.1. Experimental Setting

In the experiments, we use a personal computer configured with an Intel i7-10700 CPU, a NVIDIA RTX 2080 graphics card, and 32 GB of memory. PyTorch1.11.0 is used to implement all methods.

Following [13], we select 30 classes of images from the original ImageNet database and scale them to the size of 96 × 96 to obtain the Tiny-ImageNet as the dataset for our experiment. Specifically, the training set contains 38,766 images and the test set includes 1500 images, while each class has 50 images. Since [20] used a grayscale dataset, all images in this experiment received grayscale processing to facilitate performance comparison.

For a fair performance evaluation, firstly, we train the CSNet [20] network in this environment with images divided into 32 × 32 blocks. Then, three classic classification networks, VGG16, ResNet50, and DenseNet121, are trained with the Tiny-ImageNet dataset.

Finally, we train the proposed IPPARNet model with the pre-trained CSNet network and the three classification networks. While training, we fix the parameters of the classifiers and alternately train the discriminator network D and the adversarial network Adv-G. Playing the two-player minimax game, the discriminator D can prompt the adversarial reconstruction network Adv-G to generate adversarial reconstructed images that are more similar to the original images. At the same time, the adversarial reconstruction network Adv-G and the restoration network R are jointly trained to obtain better restoration of the sanitized images.

The optimization algorithm Adam is employed and the batch size is 32. The learning rate of adversarial network Adv-G, recovery network R, and discriminator D is set to 0.0001, which is reduced to 0.1 times every 50 rounds. When the sampling rate is 0.1, the hyperparameters are set as follows: $g_{1}$ = 0.001, $g_{2}$ = 0.001, $g_{3}$ = 0.0005, $r_{1}$ = 0.001, and α = 2.

### 4.2. Results and Analysis

#### 4.2.1. Benchmark

We not only take account of the quality of the CS reconstructed images and the ability to fool the DNN models, but also have thought to reverse the adversarial reconstructed images to sanitized samples. However, to the best of our knowledge, there is no previous study dealing with the above tasks. Therefore, we use the reconstructed images ${X\prime }_{\mathrm{CSNet}}$ from the CSNet as the benchmark, then evaluate the performance of the adversarial reconstructed images X′ and the restored sanitized images X″. Specifically, X, $\tilde{X}$, X′, and X″ that appear in the following paper represent the set of all $x_{i}$, ${\tilde{x}}_{i}$, $x_{i}^{\prime }$, and $x_{i}^{\prime \prime }$ on the test set, respectively.

For evaluation, we use 1500 grayscale images of size 96 × 96 from the Tiny-ImageNet test set and employ peak signal-to-noise ratios (PSNRs) and recognition accuracy as evaluation metrics.

At first, we test the performance of the pre-trained VGG16, ResNet50, and DenseNet121 classifiers for 1500 Tiny-ImageNet test images. Table 1 shows the recognition accuracy of the original test set on the three classification networks. The row of “Average” means the average recognition rate of the three classifiers. Compared with the trained one on all ImageNet database, the recognition rate of the three recognition networks we trained is much lower. This is because our training set is much less than the ImageNet database, accounting for less than 1/30. Therefore, getting relatively low recognition rate is a reasonable phenomenon.

Then, setting the sampling rate as 0.1, 0.2, 0.3 and 0.5, we employ the CSNet to implement reconstruction for the test set of the Tiny-ImageNet. Table 2 shows the image quality of the CSNet reconstructed images under different sampling rates with the metric PSNR. Table 3 shows the recognition rates of VGG16, ResNet50, and DenseNet121 for ${X\prime }_{\mathrm{CSNet}}.$

One can see from Table 2 and Table 3 that with the increase in sampling rate, the PSNR values and recognition accuracy of CSNet reconstructed images ${X\prime }_{\mathrm{CSNet}}$ were improved. Compared with the original image, at different sampling rates, the recognition accuracy of the reconstructed image ${X\prime }_{CSNet}$ on the three classifiers decreased to varying degrees. However, even when the sampling rate is 0.1, the reconstructed images ${X\prime }_{\mathrm{CSNet}}$ can achieve a recognition accuracy of 41–61% on the three classifiers. While the sampling rate is 0.5, their recognition accuracy is almost equal to that achieved on the original images. This means that malicious users can acquire a lot of sensitive information from CSNet reconstructed images, ${X\prime }_{\mathrm{CSNet}}$, which poses a great privacy threat.

#### 4.2.2. Performance Evaluation

The goal of the proposed IPPARNet is to take the machine recognition metric into account while retaining good visual quality. On the one hand, the adversarial reconstructed images X′ can mislead the target classifiers, making it difficult for semi-authorized users to abuse them effectively, so as to achieve the goal of privacy protection. On the other hand, authorized users can restore sanitized images X″ from X′, and the recognition accuracy can be improved as high as possible, which is helpful for the machine’s subsequent recognition tasks. That is to say, while keeping a reasonable PSNR, for the adversarial reconstructed images X′, the lower the recognition rate, the better the performance. However, for the sanitized images X″, the higher the recognition rate, the better the performance.

1. Analysis of Recognition Accuracy

At different sampling rates, the recognition rates of VGG16, ResNet50, and DenseNet121 classifiers for adversarial reconstructed images X′ and sanitized images X″ are shown in Table 4. It can be observed that for each sampling rate, the recognition rates of the adversarial reconstructed images X′ are significantly lower than that of the CSNet reconstructed images ${X\prime }_{\mathrm{CSNet}}$. Take the sampling rate of 0.1 as an example. The recognition rates of X′, which is generated by our adversarial reconstruction network, on the three classifiers VGG16, ResNet50, and DenseNet121 are not more than 1/10, which are 6.0%, 10.0%, and 7.6%, respectively. Compared with ${X\prime }_{\mathrm{CSNet}}$, the recognition rates of X′ are relatively reduced by 85.3%, 83.6%, and 86.2%. When setting the sampling rate as 0.2, 0.3, and 0.5, the average recognition rate of adversarial reconstructed images X′ on the three classifiers drops from 71.5%, 74.0%, and 78.6% to 8.8%, 12.8%, and 13.5%, respectively. It can be seen that the relative declines are all greater than 82.0%, which results in semi-authorized machine users being unable to recognize these adversarial reconstructed images precisely. In other words, semi-authorized machine users are prevented from performing effective data analysis and model training tasks, and the privacy of images is protected.

However, authorized users can obtain sanitized reconstructed images X″ with the additional restoration network. When the sampling rate is 0.1, the corresponding recognition rates on the three classifiers are 49.2%, 62.0%, and 55.6%, respectively, which are slightly higher than the recognition rates of ${X\prime }_{\mathrm{CSNet}}$ reconstructed by the CSNet. At other sampling rates, the average recognition rates of the sanitized reconstructed images X″ on the three classifiers can reach 69.6%, 70.3%, and 74.7%, which are approximate to that achieved by ${X\prime }_{\mathrm{CSNet}}$. Obviously, X″ contributes to the machine recognition tasks.

2. Analysis of Image Visual Quality

Table 5 shows the PSNR values of the CSNet reconstructed images ${X\prime }_{\mathrm{CSNet}}$, the adversarial reconstructed images X′, and the sanitized images X″. Take the sampling rate of 0.1 as an example. Compared with ${X\prime }_{\mathrm{CSNet}}$, the PSNR value of our adversarial reconstructed images X′ is only reduced by 0.39 dB. At different sampling rates, the PSNR values drops by 0.2–6.1%. When the sampling rate is 0.5, the PSNR value remains at 27.82 dB, which still provides a good visual effect for human eyes. In comparison, the PSNR values of the sanitized images X″ have a smaller decrease. In the case of sampling rate 0.1, it is only 0.06 dB lower than ${X\prime }_{\mathrm{CSNet}}$. Since the PSNR value of X″ is greater than that of X′ at the same sampling rate, we infer that X″ has better visual quality than X′.

**Figure 3:**
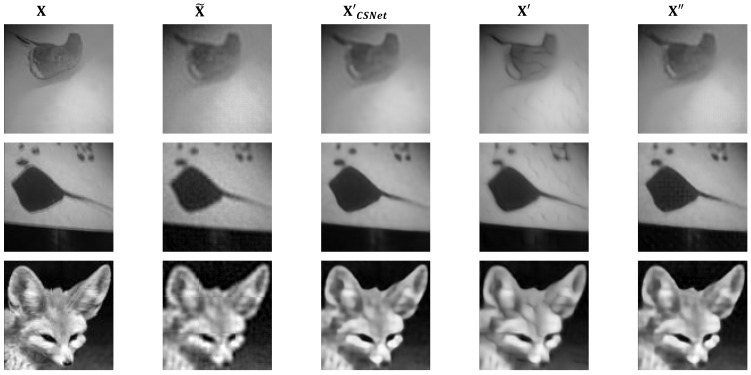
Visual quality comparisons of three images from Tiny-ImageNet. X, $\tilde{X}$, ${X\prime }_{\mathrm{CSNet}}$,  X ′ and X″ at the sampling rate 0.1 are indicated from the first to the fifth column, respectively.

To perceive the visual quality of the image intuitively, three images from the Tiny-ImageNet test set were randomly selected as representatives. Figure 3 illustrates the original images X, the corresponding initial reconstructed images $\tilde{X}$, the reconstructed images ${X\prime }_{\mathrm{CSNet}}$ of CSNet, our adversarial reconstructed images X′, and the restored sanitized images X″ of the three images.

It can be observed that our adversarial reconstructed images X′ are not disturbed significantly compared with CSNet reconstructed images ${X\prime }_{\mathrm{CSNet}}$ and the sanitized images X″ restored by authorized users are visually indistinguishable from ${X\prime }_{\mathrm{CSNet}}$. Both X′ and X″ have good visual quality for human beings and X″ is better.

During the reconstruction, the network I_Rec of the CSNet learns a linear mapping to obtain relatively good initial reconstructed images, $\tilde{X}$. Then, with a nonlinear network D_Rec, the residual between the initial reconstructed images $\tilde{X}$ and the original images X is learnt, which can eliminate the block artifacts of $\tilde{X}$ and further improve the visual quality simultaneously. However, the adversarial reconstruction network Adv-G aims to learn a perturbation that makes the final reconstructed images have the ability to deceive DNN models with this nonlinear network. That is, by adding the perturbation, the final reconstructed images X″ can induce the recognizer to make wrong judgments while ensuring it has good visual quality.

**Figure 4:**
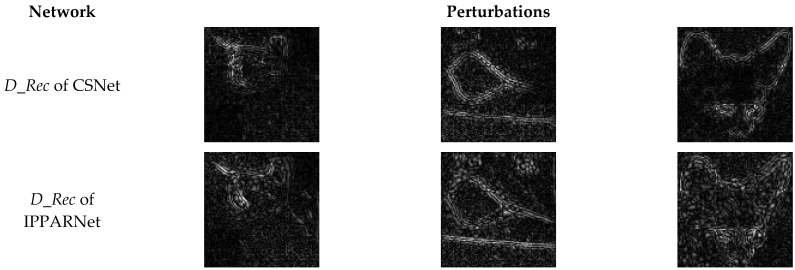
Comparisons of perturbation generated by *D_Rec* networks.

Figure 4 shows the perturbations learned by the network D_Rec of CSNet and the proposed IPPARNet. It can be seen that in our method, the learned perturbations cannot only eliminate block artifacts and supplement the contour details, but also acquire additional adversarial perturbations.

In summary, with the proposed IPPARNet, under the premise of ensuring good visual quality, the recognition rate of the adversarial reconstructed images X′ can be reduced by more than 82% compared with CSNet reconstructed images ${X\prime }_{\mathrm{CSNet}}$, while authorized users can restore sanitized images  X ″ which achieve the approximate recognition accuracy of the ${X\prime }_{\mathrm{CSNet}}$.

## 5. Conclusions

In this paper, we propose a novel image CS reconstruction method that supports inversible privacy protection. On the one hand, we use adversarial samples as a weapon of privacy protection. In the process of image CS reconstruction, we simultaneously consider the visual quality of the reconstructed image and its ability to deceive the DNN models. On the other hand, we jointly train both the adversarial reconstruction network and the restoration network to ensure the invertibility of adversarial reconstructed images. Numerous experimental results show that our method can generate adversarial reconstructed images with a high attack success rate and sanitized reconstructed images with better recognition accuracy, while maintaining good visual quality. The adversarial reconstructed images can protect private data from malicious users, while the sanitized reconstructed images can safeguard the legitimate rights of authorized users and avoid unnecessary resource wastage. In future work, we will explore how to further improve the recognition accuracy of the sanitized reconstructed images.

## Figures and Tables

**Table 1:** Recognition rates of VGG16, ResNet50, and DenseNet121 for original images of Tiny-ImageNet dataset (%).

	Testing Set	Tiny-ImageNet
Recognizer		
VGG16		81.4
ResNet50		80.2
DenseNet121		77.2
Average		79.5

**Table 2:** Evaluating image quality of ${X\prime }_{\mathrm{CSNet}}$ in terms of PSNR (dB).

Sampling Rate	PSNR
0.1	26.33
0.2	29.19
0.3	30.30
0.5	33.27

**Table 3:** Recognition rates of VGG16, ResNet50, and DenseNet121 for ${X\prime }_{\mathrm{CSNet}}$ (%).

	Sampling Rate	0.1	0.2	0.3	0.5
Recognizer					
VGG16		40.8	69.8	70.8	79.6
ResNet50		61.0	73.4	74.4	79.4
DenseNet121		55.2	71.2	76.8	77.0
Average		52.3	71.5	74.0	78.6

**Table 4:** Comparing recognition rates of three recognizers for ${X\prime }_{\mathrm{CSNet}}$, X′ and X″ at various sampling rates (%).

	Sampling Rate	0.1	0.2	0.3	0.5
Images					
${X\prime }_{\mathrm{CSNet}}$	Average	52.3	71.5	74.0	78.6
X′	VGG16	6.0	7.2	11.6	10.4
	ResNet50	10.0	12.8	15.2	19.0
	DenseNet121	7.6	6.4	11.6	11.2
	Average	7.8	8.8	12.8	13.5
X″	VGG16	49.2	68.8	69.8	74.8
	ResNet50	62.0	72.0	72.8	76.8
	DenseNet121	55.6	68.0	68.4	72.4
	Average	55.6	69.6	70.3	74.7

**Table 5:** Comparing image quality of ${X\prime }_{\mathrm{CSNet}}$, X′ and X″ in terms of PSNR (dB).

	Sampling Rate	0.1	0.2	0.3	0.5
Images					
${X\prime }_{\mathrm{CSNet}}$		26.33	29.19	30.30	33.27
X′		25.94	27.52	27.78	27.82
X″		26.27	28.91	29.09	31.25

## Data Availability

All experiments from this paper are performed on public datasets.

## Abbreviations

The following abbreviations are used in this manuscript:

CS	Compressed sensing
OMP	Orthogonal matching pursuit
GPSR	Gradient projection for sparse reconstruction
DNN	Deep neural network
BP	Basis pursuit
MP	Matching pursuit
SDA	Stacked denoising autoencoder
CNN	Convolutional neural network
BCS	Block compressed sensing
FGSM	Fast gradient sign method
BIM	Basic iterative method
PGD	Projected gradient descent
GAN	Generative adversarial networks
ReLU	Rectified linear unit
